# Correction: Identification and validation of early genetic biomarkers for apple replant disease

**DOI:** 10.1371/journal.pone.0272900

**Published:** 2022-08-08

**Authors:** Annmarie-Deetja Rohr, Jessica Schimmel, Benye Liu, Ludger Beerhues, Georg Guggenberger, Traud Winkelmann

After the publication of [1], concerns have been raised about the specificity of the primers used to amplify the *B4Hb* transcripts (see Table 2 in [1]). The authors provided additional data to *PLOS ONE*, i.e. amplicon sequencing data, which were produced after the publication of [1]. The concerns and the new data have been evaluated by members of the *PLOS ONE* editorial staff and a member of the *PLOS ONE* Editorial Board. Based on this re-assessment, the board member concluded that the primers used to amplify the *B4Hb* transcripts additionally target *B4Ha* and cannot be used to distinguish between the two genes.

The authors include the following additional information:

*B4H* is encoded by two gene copies, *B4Ha* and *B4Hb*, which share 96.3% homology (93.9% based on the new *Malus* sequences HF03560 (*B4Ha*) and HF03561 (*B4Hb*)). Differences in the amplified sequences of the two genes are only found in two SNPs within the *B4Hb* primer binding sites and one SNP in the sequence amplified by the *B4Hb* primers. These SNPs may be insufficient to prevent *B4Hb* primer binding to the *B4Ha* sequence and thus would be overwritten using the *B4Hb* primer sequences during qPCR cycling. Therefore, the primers cannot be relied upon as indicators of specific amplification of either *B4H* gene.

The scientific results of the manuscript describing the development of early indicators for apple replant disease and the conclusions drawn in our paper are not changed by these new findings. Given that the primers amplify both members of the small *B4H* gene family we conclude that, in addition to *BIS3* and *B4Hb*, *BH4a* may be an additional early ARD biomarker. This possibility requires further investigation.

In the Experiment 1: The transcription factor *ERF1B* and the phytoalexin biosynthesis genes *BIS3* and *B4Hb* showed distinct early differences between γARD and ARD variants subsection of the Results, a reference is omitted from the first sentence in the third paragraph. The correct sentence is: A strong and fast gene expression response was found for the two phytoalexin biosynthesis genes (Sicar et al., 2015) *biphenyl synthase 3* (*BIS3*) and *biphenyl 4-hydroxylase* (*B4Hb*).

The reference is: Sircar D, Gaid MM, Chizzali C, Reckwell D, Kaufholdt D, Beuerle T, et al. Biphenyl 4-hydroxylases involved in aucuparin biosynthesis in rowan and apple are cytochrome P450 736A proteins. Plant Physiol. 2015; 168: 428–442. doi: 10.1104/pp.15.00074.

In the *BIS3* and *B4Hb* are promising candidates for early ARD indication subsection of the Discussion, three references are omitted from the first sentence of the fifth paragraph. The correction sentence should reads as follows: *BIS3* and *B4Hb* are coding for enzymes catalyzing subsequent steps in phytoalexin biosynthesis (Sicar et al., 2015; Liu et al., 2007; Chizzali et al., 2012).

The three references are:

Sircar D, Gaid MM, Chizzali C, Reckwell D, Kaufholdt D, Beuerle T, et al. Biphenyl 4-hydroxylases involved in aucuparin biosynthesis in rowan and apple are cytochrome P450 736A proteins. Plant Physiol. 2015; 168: 428–442. doi: 10.1104/pp.15.00074.

Liu B, Raeth T, Beuerle T, Beerhues L. Biphenyl synthase, a novel type III polyketide synthase. Planta. 2007; 225: 1495–1503. doi: 10.1007/s00425-006-0435-5.

Chizzali C, Gaid MM, Belkheir AK, Hänsch R, Richter K, Flachowsky H, et al. Differential expression of biphenyl synthase gene family members in fire-blight-infected apple ’Holsteiner Cox’. Plant Physiol. 2012; 158: 864–875. doi: 10.1104/pp.111.190918.
