# Supplementary figures and images for: Identification and validation of early genetic biomarkers for apple replant disease
Source: PLoS One. 2020 Sep 24;15(9):e0238876. doi: 10.1371/journal.pone.0238876 (PMC7514092; doi:10.1371/journal.pone.0238876)

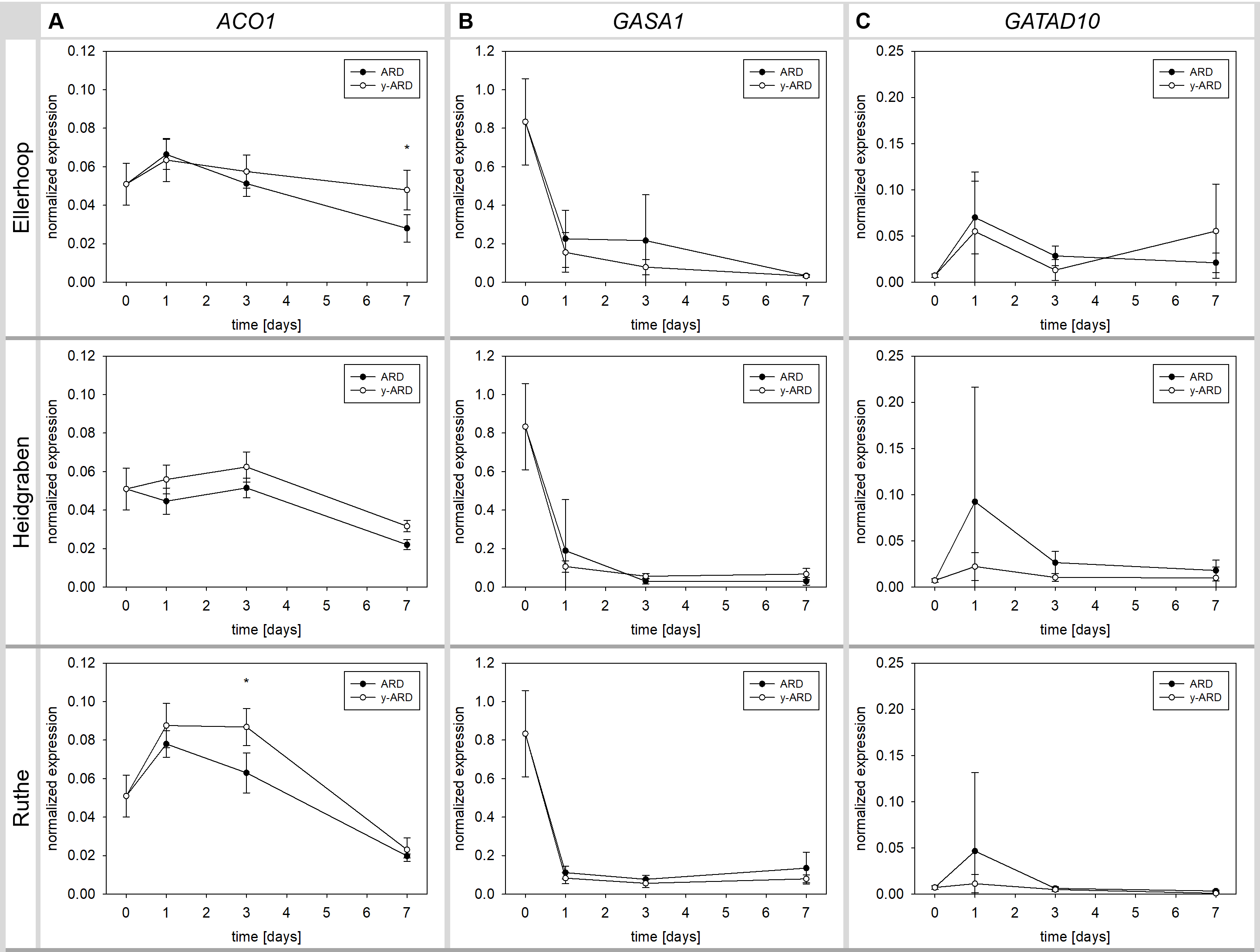

Supplement: S1 Fig — Means ± SD, n = 4 pooled samples. Significant differences at each time point shown for p < 0.05 (*), p < 0.01 (**) and p < 0.001 (***) as investigated by Tukey tests. (PNG) [file pone.0238876.s001.png]

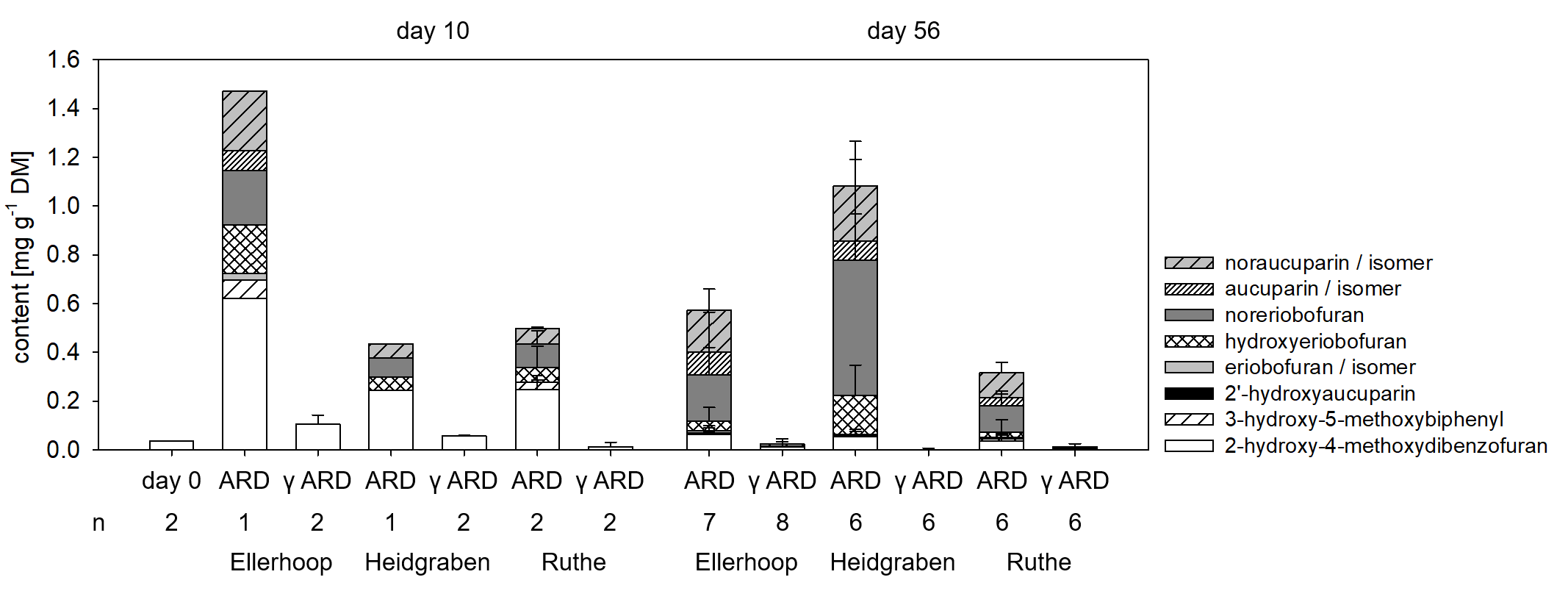

Supplement: S2 Fig — Number of root samples analyzed indicated by n. (PNG) [file pone.0238876.s002.png]

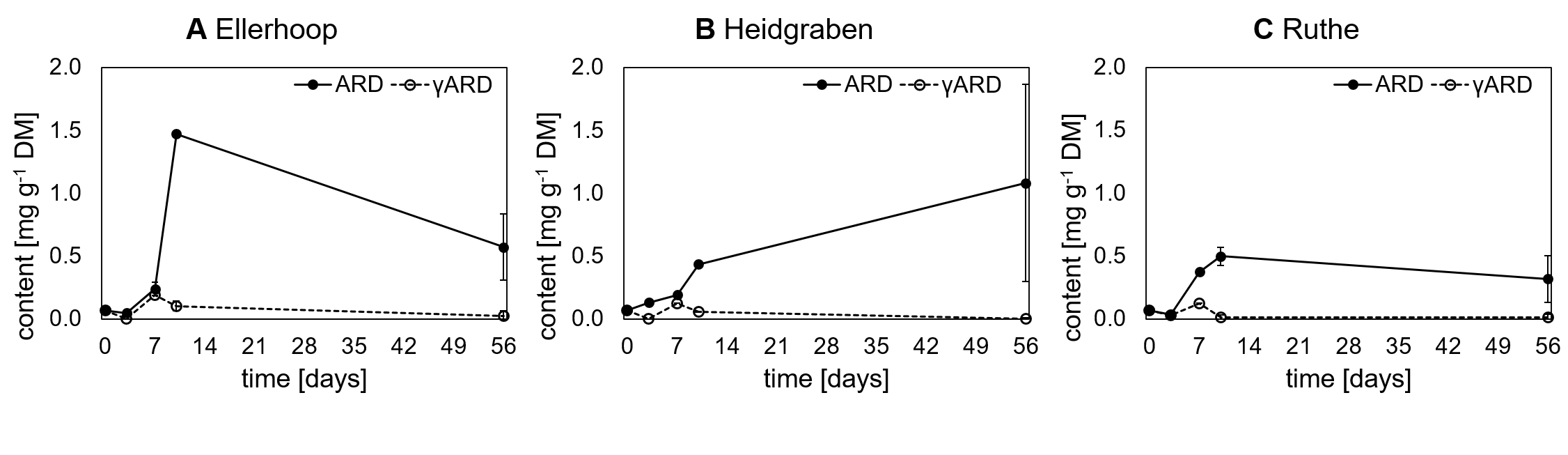

Supplement: S3 Fig — Number of samples analyzed is indicated in S4 Table. (PNG) [file pone.0238876.s003.png]

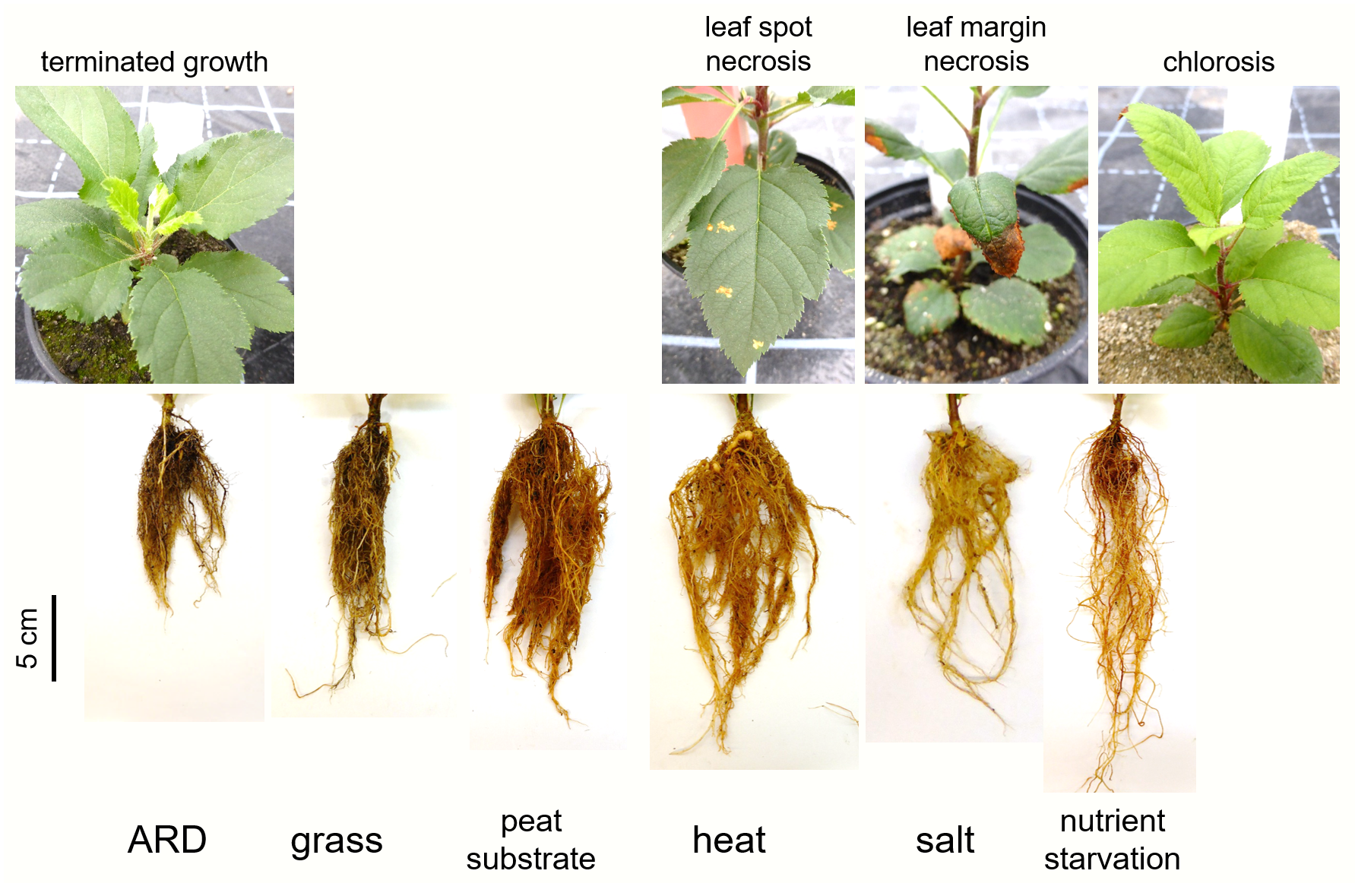

Supplement: S4 Fig — See Table 1 for details on the variants. (PNG) [file pone.0238876.s004.png]

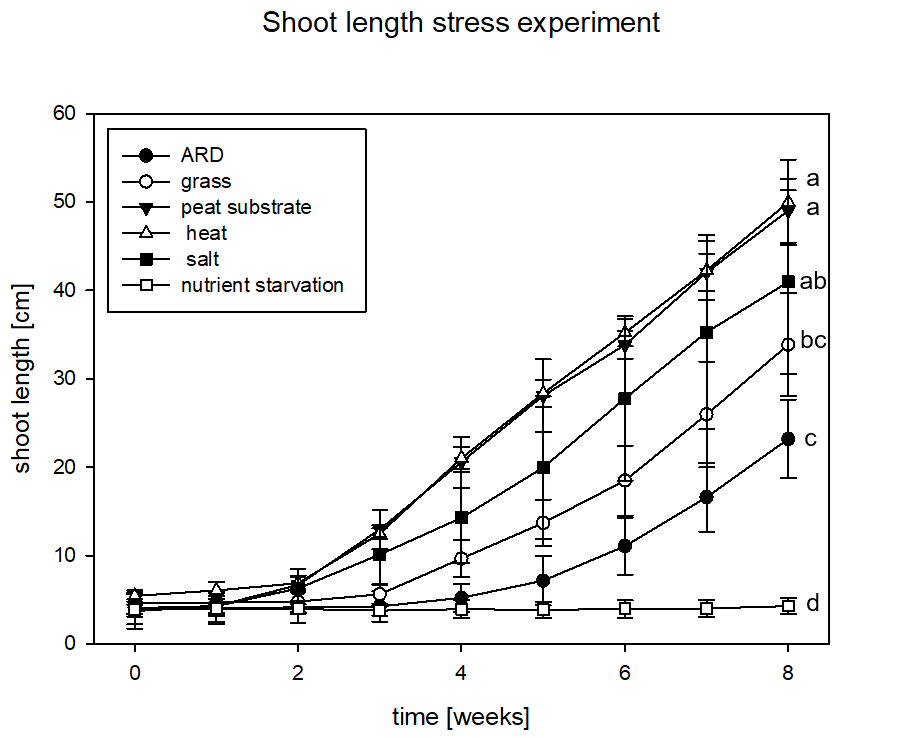

Supplement: S5 Fig — Different letters indicate a statistical difference (Tukey Test, p < 0.05) between variants. See Table 1 for details on the variants. (PNG) [file pone.0238876.s005.png]
