# Supplementary material for: Identification and validation of early genetic biomarkers for apple replant disease
Source: PLoS One. 2020 Sep 24;15(9):e0238876. doi: 10.1371/journal.pone.0238876 (PMC7514092; doi:10.1371/journal.pone.0238876)
Supplement: S2 Table — (DOCX) [file pone.0238876.s007.docx]

**S2 Table: Information on biological and technical replicates of experiment 1.**

|  |  |  | Gene expression analysis | | | | | | Phytoalexin analysis | | | | | | | |
| --- | --- | --- | --- | --- | --- | --- | --- | --- | --- | --- | --- | --- | --- | --- | --- | --- |
| Variant | Substrate / soil | Treatment /  specifications | Number  of plants | | | Number of pooled  samples analyzed (n) | | | Number  of plants | | | | Number of pooled  samples analyzed (n) | | | |
| day 0 | Peat substrate | none | 20 | | | 4 | | | 30 | | | | 2 | | | |
|  |  |  | day1 | day3 | day7 | day1 | day3 | day7 | day3 | day7 | day10 | day56 | day3 | day7 | day10 | day56 |
| ARD Ellerhoop | ARD soil Ellerhoop | fertilized | 20 | 20 | 20 | 4 | 4 | 4 | 10 | 10 | 10 | 10 | 1 | 2 | 1 | 7 |
| γARD Ellerhoop | ARD soil Ellerhoop | γ-irradiated, fertilized | 20 | 20 | 20 | 4 | 4 | 4 | 10 | 10 | 10 | 10 | 1 | 1 | 2 | 8 |
| ARD Heidgraben | ARD soil Heidgraben | fertilized | 20 | 20 | 20 | 4 | 4 | 4 | 10 | 10 | 10 | 10 | 1 | 2 | 1 | 6 |
| γARD Heidgraben | ARD soil Heidgraben | γ-irradiated, fertilized | 20 | 20 | 20 | 4 | 4 | 4 | 10 | 10 | 10 | 10 | 1 | 1 | 2 | 6 |
| ARD Ruthe | ARD soil Ruthe | fertilized | 20 | 20 | 20 | 4 | 4 | 4 | 10 | 10 | 10 | 10 | 1 | 1 | 2 | 6 |
| γARD Ruthe | ARD soil Ruthe | γ-irradiated, fertilized | 20 | 20 | 20 | 4 | 4 | 4 | 10 | 10 | 10 | 10 | 1 | 1 | 2 | 6 |
|  |  | **total** | **380** | | | **76** | | | **270** | | | | **63** | | | |
