# Supplementary material for: Identification and validation of early genetic biomarkers for apple replant disease
Source: PLoS One. 2020 Sep 24;15(9):e0238876. doi: 10.1371/journal.pone.0238876 (PMC7514092; doi:10.1371/journal.pone.0238876)
Supplement: S3 Table — (DOCX) [file pone.0238876.s008.docx]

**S3 Table: Information on biological and technical replicates of experiment 2.**

|  |  |  | Gene expression analysis | | | | Phytoalexin analysis | Nutrient analysis |
| --- | --- | --- | --- | --- | --- | --- | --- | --- |
| Variant | Substrate / soil | Treatment /  specifications | Number  of plants | | Number of pooled  samples analyzed (n) | | Number  of plants = number of samples analyzed (n) | Number  of plants = number of samples analyzed (n) |
| day 0 | peat  substrate | none | 15 | | 5 | | 5 | 0 |
|  |  |  | day7 | day14 | day7 | day14 | Day14 | day56 |
| ARD | ARD soil Heidgraben | fertilized | 15 | 15 | 5 | 5 | 5 | 5 |
| Grass | control soil Heidgraben | fertilized | 15 | 15 | 5 | 5 | 5 | 5 |
| Peat (substrate) | peat substrate + quartz sand (2 + 1) | fertilized | 15 | 15 | 5 | 5 | 5 | 5 |
| Heat | peat substrate + quartz sand (2 + 1) | fertilized, plants 3 days  at 37°C | 15 | 15 | 5 | 5 | 5 | 5 |
| Salt | peat substrate + quartz sand (2 + 1) | one-time application of 50 mL 0.17 M NaCl (10 g L^-1^) at the start of the experiment, fertilized | 15 | 15 | 5 | 5 | 5 | 5 |
| Nutrient starvation | quartz  sand | not fertilized | 15 | 15 | 5 | 5 | 5 | 4 |
| 30 |  | **total** | **195** | | **65** | | **35** | **29** |
