# Supplementary material for: Identification and validation of early genetic biomarkers for apple replant disease
Source: PLoS One. 2020 Sep 24;15(9):e0238876. doi: 10.1371/journal.pone.0238876 (PMC7514092; doi:10.1371/journal.pone.0238876)
Supplement: S4 Table — (DOCX) [file pone.0238876.s009.docx]

S4 Table: Pooling and masses of freeze-dried root material from experiment 1 for phytoalexin analysis.

| Day | ARD soil | n  (number of pooled samples) | Pool number  per variant | Number of individual root systems united | Dry mass submitted to analysis [mg] |
| --- | --- | --- | --- | --- | --- |
| 0 | - | 2 | 1 | 15 | 133.9 |
| 0 | - |  | 2 | 15 | 105.0 |
| 3 | Ruthe ARD | 1 | 1 | 10 | 86.2 |
| 3 | Ruthe γARD | 1 | 1 | 10 | 68.5 |
| 3 | Heidgraben ARD | 1 | 1 | 10 | 111.3 |
| 3 | Heidgraben γARD | 1 | 1 | 10 | 74.2 |
| 3 | Ellerhoop ARD | 1 | 1 | 10 | 106.7 |
| 3 | Ellerhoop γARD | 1 | 1 | 10 | 65.5 |
| 7 | Ruthe ARD | 1 | 1 | 10 | 84.3 |
| 7 | Ruthe γARD | 1 | 1 | 10 | 120.7 |
| 7 | Heidgraben ARD | 2 | 1 | 4 | 77.5 |
| 7 | Heidgraben ARD |  | 2 | 6 | 59.7 |
| 7 | Heidgraben γARD | 1 | 1 | 10 | 97.0 |
| 7 | Ellerhoop ARD | 2 | 1 | 5 | 61.6 |
| 7 | Ellerhoop ARD |  | 2 | 5 | 73.0 |
| 7 | Ellerhoop γARD | 1 | 1 | 10 | 87.6 |
| 10 | Ruthe ARD | 2 | 1 | 5 | 82.0 |
| 10 | Ruthe ARD |  | 2 | 5 | 79.3 |
| 10 | Ruthe γARD | 2 | 1 | 5 | 51.4 |
| 10 | Ruthe γARD |  | 2 | 5 | 69.5 |
| 10 | Heidgraben ARD | 1 | 1 | 10 | 92.9 |
| 10 | Heidgraben γARD | 2 | 1 | 6 | 71.1 |
| 10 | Heidgraben γARD |  | 2 | 4 | 42.2 |
| 10 | Ellerhoop ARD | 1 | 1 | 10 | 163.6 |
| 10 | Ellerhoop γARD | 2 | 1 | 4 | 91.2 |
| 10 | Ellerhoop γARD |  | 2 | 6 | 75.2 |

| Day | ARD soil | n  (number of pooled samples) | Pool number  per variant | Number of individual root systems united | Dry mass submitted to analysis [mg] |
| --- | --- | --- | --- | --- | --- |
| 56 | Ruthe ARD | 6 | 1 | 1 | 101.0 |
| 56 | Ruthe ARD |  | 2 | 1 | 113.1 |
| 56 | Ruthe ARD |  | 3 | 1 | 113.7 |
| 56 | Ruthe ARD |  | 4 | 1 | 116.0 |
| 56 | Ruthe ARD |  | 5 | 1 | 109.9 |
| 56 | Ruthe ARD |  | 6 | 1 | 107.0 |
| 56 | Ruthe γARD | 6 | 1 | 1 | 107.0 |
| 56 | Ruthe γARD |  | 2 | 1 | 106.6 |
| 56 | Ruthe γARD |  | 3 | 1 | 112.8 |
| 56 | Ruthe γARD |  | 4 | 1 | 136.0 |
| 56 | Ruthe γARD |  | 5 | 1 | 107.1 |
| 56 | Ruthe γARD |  | 6 | 1 | 136.2 |
| 56 | Heidgraben ARD | 6 | 1 | 1 | 111.0 |
| 56 | Heidgraben ARD |  | 2 | 1 | 107.6 |
| 56 | Heidgraben ARD |  | 3 | 1 | 107.6 |
| 56 | Heidgraben ARD |  | 4 | 1 | 130.9 |
| 56 | Heidgraben ARD |  | 5 | 1 | 107.4 |
| 56 | Heidgraben ARD |  | 6 | 1 | 107.3 |
| 56 | Heidgraben γARD | 6 | 1 | 1 | 103.6 |
| 56 | Heidgraben γARD |  | 2 | 1 | 108.8 |
| 56 | Heidgraben γARD |  | 3 | 1 | 108.8 |
| 56 | Heidgraben γARD |  | 4 | 1 | 135.2 |
| 56 | Heidgraben γARD |  | 5 | 1 | 111.3 |
| 56 | Heidgraben γARD |  | 6 | 1 | 159.0 |
| 56 | Ellerhoop ARD | 7 | 1 | 1 | 99.6 |
| 56 | Ellerhoop ARD |  | 2 | 1 | 102.2 |
| 56 | Ellerhoop ARD |  | 3 | 1 | 98.0 |
| 56 | Ellerhoop ARD |  | 4 | 1 | 104.8 |
| 56 | Ellerhoop ARD |  | 5 | 1 | 136.6 |
| 56 | Ellerhoop ARD |  | 6 | 1 | 68.9 |
| 56 | Ellerhoop ARD |  | 7 | 1 | 103.1 |
| 56 | Ellerhoop ARD |  | 8 | 1 | 111.6 |
| 56 | Ellerhoop γARD | 8 | 1 | 1 | 108.9 |
| 56 | Ellerhoop γARD |  | 2 | 1 | 91.0 |
| 56 | Ellerhoop γARD |  | 3 | 1 | 102.9 |
| 56 | Ellerhoop γARD |  | 4 | 1 | 102.2 |
| 56 | Ellerhoop γARD |  | 5 | 1 | 134.7 |
| 56 | Ellerhoop γARD |  | 6 | 1 | 101.0 |
| 56 | Ellerhoop γARD |  | 7 | 1 | 125.0 |
| 56 | Ellerhoop γARD |  | 8 | 1 | 116.2 |
