# Supplementary material for: Identification and validation of early genetic biomarkers for apple replant disease
Source: PLoS One. 2020 Sep 24;15(9):e0238876. doi: 10.1371/journal.pone.0238876 (PMC7514092; doi:10.1371/journal.pone.0238876)
Supplement: Attachment — Submitted filename: Response to Reviewers.docx [file pone.0238876.s010.docx]

PONE-D-20-08054
Identification and validation of early genetic biomarkers for apple replant disease
PLOS ONE

Editor: Suprasanna Penna (Academic Editor)

Dear Dr. Suprasanna Penna, dear Dr. Yanmin Zhu, dear unknown reviewer, dear Dr. R.S. Bhat,

we are very grateful for your valuable time spent into our manuscript and your comments which we carefully considered and followed wherever possible as explained below.

Please find our responses indicated in blue below each point raised by one of you.

We hope that you will find all points addressed in the right way and look forward to hearing from you again soon.

Thank you and kind regards

Annmarie-Deetja Rohr and Traud Winkelmann

**REVISION NOTES / DEADLINE 23.07.2020**

**Reviewer 1**

Ln 128-129: “In the grassland plots within the reference sites, no members of the Rosaceae had been grown, thus, this soil served as a control soil not affected by replant disease”. Need to be cautious that some members in this pathogen complex are not specific to ARD, rather they are the wide-spectrum opportunistic necrotrophic attackers. That is why you observed the induction of the target gene at low level.

Thank you for pointing that out. Indeed, this is also one of our explanations for the observed elevated gene expression in the grass variant! In addition to that, we are considering that the phytoalexin response itself is not fully specific to ARD but boosted by it. We changed the passage accordingly: “In the grassland plots within the reference sites, no members of the Rosaceae had been grown. Thus, this soil served as a control soil that was not affected by replant disease but contained microorganism communities native to the site.” (ln. 127 ff. of the revised manuscript)

ln 166-167: “Cultivation during the first three days was carried out without additional lighting. From then on, additional light was provided by SON-T Philips Master Agro 400 W lamps”. Why extra light is required? Is this a random event or special design?

We were intentionally providing extra light especially in the winter months when solar radiation was not high enough to provide optimal growing conditions. The additional light is automatically activated if the solar radiation is not sufficient (“when solar radiation fell below 25 klx to provide 16 h of daylight”).

The text was edited for more clarity (ln. 167-170): “From then on, additional light was provided by SON-T Philips Master Agro 400 W lamps (Hamburg, Germany) if solar radiation fell below 25 klx to provide 16 h of daylight and thus comparable growing conditions over the whole year.”

Ln 306-307: “In soil from Ellerhoop, shoot length did not differ significantly from week 7 on and individual plants even had a greater shoot length in ARD soil compared to γARD.” What is the speculation about this observation?

We agree that this needs explanation. We speculate that either spores of pathogenic microorganisms might have survived gamma irradiation or that soil chemical or physical soil factors might have caused a generally reduced growth of our apple plantlets. We included this in ln. 545 ff.: “This could point to the fact that either chemical or physical characteristics of this soil caused slower growth, beneficial soil organisms were lacking or pathogens survived the irradiation and thus occurred in the γ-irradiated soil. The latter speculation is supported by the observation that roots grown in γ-irradiated Ellerhoop soil contained significantly higher phytoalexin contents compared to the γ-irradiated soils of the other two sites investigated (Fig 4).”

Ln 372-373: “In soils from Ellerhoop and Ruthe, the total phytoalexin contents increased rapidly during day 7 and day 10, decreased at day 56…” what is the speculation of the decrease?

Thank you for raising this point. At the early time points we had only little root material to work with, which unfortunately led to low replicate numbers for the analysis. Therefore, the soundness of the data for the early time points is somewhat limited. To account for this, we changed this passage accordingly: “In all three soils, the total phytoalexin contents increased rapidly during the initial days they were still high at day 56.“

In Figure 4, any comment or speculation on why the second site induce the most increase of secondary metabolite production?

One might speculate that the incidence of ARD was extremely strong for this site. This might be due to a high concentration of pathogenic organisms or their specific combination at the soil from Heidgraben since also plant growth was more severely affected here (Fig. 2).

In Fig 6, the expression of these genes was obviously higher in Control soil Heidgraben (grass), obviously higher than “Peat substrate + quartz sand”, does this mean other soil microorganisms, which may not be pathogenic to apple root, triggered the induction of these genes? If this is the case, then ARD pathogens are not the only microorganisms induce the gene expression, rather many other soilborne necrotrophs has the same effect on the expression of these genes.

Thank you for raising this important point. Indeed, all three genes showed a response also towards grass soil as you pointed out. We believe that this induction was caused by microorganisms already present in the healthy soil (grass soil in our experiment) and discuss in section 4.3 how this can be linked to the ARD etiology via a feedback-loop. In ln 662 – 684 of the revised manuscript we state “With the comparison to grass soil, we have now proven that pathogens present in this control soil also induce the expression of our candidate genes *BIS3, B4Hb* and *ERF1B* to a certain extent (Fig. 6).“

To clarify that *BIS3* and *B4Hb* also respond to healthy soil, we added in ln 623 ff. “This unspecific response is important to consider regarding the limitations of the explanatory power as expression markers but may also give further insights into the ARD etiology as discussed below.”

Ln 528-529: “This could point to the fact that pathogens that affect plant growth were also present in the γ-irradiated soil.” Is it possible this treatment does not eliminate all the pathogenic factors? or alternatively sometimes a growth promoting microorganism existed in ARD soils too.
We use very high dosages of >10 kGy for γ-irradiation, but it is reported that some microorganisms can survive this treatment. Those microorganisms are generally bacterial genera which are able to form spores. However, fungi, which are most associated with ARD in the first row are almost totally reduced as we saw from soil suspension platings. In later experiments, we performed a plating of soil bacteria and fungi habitually after irradiation to confirm its effectiveness prior to an experiment. Unfortunately, this was not yet performed for these experiments.

We are aware that the approach we chose will also eliminate possible growth-promoting microorganisms. We chose γ-irradiation to compare our results to the findings in previous studies of Weiß et al 2017.

We hope that we have addressed this issue by the changes explained above when replying to your comment for ln 306/307.

Thank you a lot for your useful and encouraging review!

**Reviewer 2**

Line 356-357, the response speed of soil in Heidgraben was not the fastest according to your supporting evidences. The expression is vague, BIS3 expression reached a plateau after 3 days, in Ruthe or all?

Thank you for this careful observation. Indeed you are right, the plateau was reached after 3 days in Ruthe and not Heidgraben. We changed the passage accordingly to “The fastest response was observed in soil from Ruthe, where the BIS3 transcript reached a plateau already after 3 days.” and added “The highest *BIS3* expression was found after 56 days in soil from Ellerhoop.” (ln 367 f.).

As author suggested, BIS1 and B4Hb can act as early biomarkers for apple replant disease and indeed significantly distinguish between ARD and disinfected ARD. However, whether they can assess different levels of ARD severity or broad-spectrum soils, not limited to just two conditions, ARD with or without. Besides, three kinds of soils may not be universal enough.

Thank you for this question. With *BIS3* and *B4Hb* identified as promising candidates, there are more experiments needed to characterize them further in the way you suggested. Especially quantitative information would be most desirable in terms of an ARD marker. We are aware that although we covered different soil textures, three soils are hardly enough to make a general statement on the suitability of these markers. However, we also found these genes induced in two further soils in our studies (ln 588 ff.). It is well possible that further stressors have an influence on the expression of these genes as we only covered the most common ones as a starting point.

We added the following paragraph to the end of section 4.2 (ln 676 f.): “Further studies including soils from more sites should follow to shed additional light on the quantitative correlation between ARD severity and candidate gene expression.”

The resolution of pictures in Fig1 and fig3 are too low.

Thank you for bringing this to our attention. As recommended by the Editor, we checked the figures using the PACE tool (<https://pacev2.apexcovantage.com/>) to meet the standards of PLOS ONE.

Thanks a lot for your careful view and suggestions to improve our manuscript!

**Reviewer 3**

World Health Organization (WHO) has defined a biomarker (Strimbu K, Tavel JA. What are biomarkers? Curr Opin HIV AIDS. 2010 Nov;5(6):463-6. doi: 10.1097/COH.0b013e32833ed177. PMID: 20978388; PMCID: PMC3078627.). What is the purpose with the authors use the term “biomarker” here?

Thank you for this important comment. We believe that the term biomarker is fitting in this context. The review by Strimbu and Tavel states that “a true definition of biomarkers includes ‘almost any measurement reflecting an interaction between a biological system and a potential hazard, which may be chemical, physical, or biological. The measured response may be functional and physiological, biochemical at the cellular level, or a molecular interaction’ ”, which also applies to fields outside of human medicine.

In our study, we are investigating the biological system apple rootstock M26 and the hazard to apple cultivation presented by apple replant disease (ARD) on the molecular level of gene expression. We are looking for an indicator of ARD that can give information on the presence of ARD early on before it is causing economic losses.

We are aware that the candidates we identified require more validation to ensure that they are reliable indicators for ARD as stated in the discussion. We hope that you can agree with this view on the biomarker definition.

Define the abbreviation “γARD”

The abbreviation γARD is referring to the γ-disinfected ARD soils as stated in ln 113 of the revised manuscript: “The untreated soil will be referred to as ARD and the γ-disinfected soil as γARD“.

What do you mean by ARD variants?

Experiment 1 included ARD soil from three different sites, each untreated (ARD) or disinfected by γ-irradiation (γARD), resulting in six variants (soil and treatment combinations) in total. The untreated ARD soils of each origin are collectively denoted as ARD variants.

How many plants were used for qRT-PCR? How were these plants grown; by sexual means or by asexual means? Was there any zygosity difference among the plants? Whether the plants grown under the two conditions were genotypically comparable or same?

For Experiment 1, root systems of 380 plants were harvested in total for RT-qPCR analysis: Twenty plants were harvested on day 0 plus 20 plants times 6 soil variants on days 1, 3, and 7. Pools of five plants each were used for RNA extraction. For Experiment 2, root systems of 195 plants were sampled in total for RT-qPCR: Fifteen plants were sampled on day 0 plus 15 times 6 variants on days 7 and 14 (this is explained in detail in ln. 174 – 183). Please also see our response to your next point and the newly included supplementary information.

All plants were propagated by asexual means, i.e. they were genetically identical clones. “For both experiments, plants of the ARD sensitive apple rootstock M26 were clonally propagated in vitro via axillary shoots […]“ (ln 139 f.).

The details on the number of plant samples, technical replicates and the biological replicates used in the study need be to more clear.

Thank you for raising this important concern. To provide this information for all readers in detail we added two supplementary tables (S2 Table and S3 Table) with the summarized information on plant numbers and samples analyzed (biological replicates), which you can see below. We added references to these tables accordingly in the text (lines 177, 180, 195, 238, 240 and 267). Furthermore, all pools that were created can be traced back in the experiment data, which we will upload to the BonaRes database (https://tools.bonares.de/submission/).

In addition to that, we added for the phytoalexin analysis (line 255 ff.): “One technical replicate was measured per sample (biological replicate) after testing the stability of the system with two injections per sample. Furthermore, technical reproducibility was ensured by repeated measurements of the alkane standards at the beginning and the end of the sequences and additionally in the middle if the sequence contained more than 40 samples. At the same time, the alkanes served to calculate the retention index (RI).”

For the nutrient analysis we added “Material from one plant each was collected, which yielded 5 biological replicates, except for salt stress, where only four plants were available (S3 Table). Nutrients in each of these samples were measured in one technical replication.” (line 265 ff.).

**S2 Table: Information on biological and technical replicates of experiment 1.**

|  |  |  | Gene expression analysis | | | | | | Phytoalexin analysis | | | | | | | |
| --- | --- | --- | --- | --- | --- | --- | --- | --- | --- | --- | --- | --- | --- | --- | --- | --- |
| Variant | Substrate / soil | Treatment /  specifications | Number  of plants | | | Number of pooled  samples analyzed (n) | | | Number  of plants | | | | Number of pooled  samples analyzed (n) | | | |
| day 0 | Peat substrate | none | 20 | | | 4 | | | 30 | | | | 2 | | | |
|  |  |  | day1 | day3 | day7 | day1 | day3 | day7 | day3 | day7 | day10 | day56 | day3 | day7 | day10 | day56 |
| ARD Ellerhoop | ARD soil Ellerhoop | fertilized | 20 | 20 | 20 | 4 | 4 | 4 | 10 | 10 | 10 | 10 | 1 | 2 | 1 | 7 |
| γARD Ellerhoop | ARD soil Ellerhoop | γ-irradiated, fertilized | 20 | 20 | 20 | 4 | 4 | 4 | 10 | 10 | 10 | 10 | 1 | 1 | 2 | 8 |
| ARD Heidgraben | ARD soil Heidgraben | fertilized | 20 | 20 | 20 | 4 | 4 | 4 | 10 | 10 | 10 | 10 | 1 | 2 | 1 | 6 |
| γARD Heidgraben | ARD soil Heidgraben | γ-irradiated, fertilized | 20 | 20 | 20 | 4 | 4 | 4 | 10 | 10 | 10 | 10 | 1 | 1 | 2 | 6 |
| ARD Ruthe | ARD soil Ruthe | fertilized | 20 | 20 | 20 | 4 | 4 | 4 | 10 | 10 | 10 | 10 | 1 | 1 | 2 | 6 |
| γARD Ruthe | ARD soil Ruthe | γ-irradiated, fertilized | 20 | 20 | 20 | 4 | 4 | 4 | 10 | 10 | 10 | 10 | 1 | 1 | 2 | 6 |
|  |  | **total** | **380** | | | **76** | | | **270** | | | | **63** | | | |

**S3 Table: Information on biological and technical replicates of experiment 2.**

|  |  |  | Gene expression analysis | | | | Phytoalexin analysis | Nutrient analysis |
| --- | --- | --- | --- | --- | --- | --- | --- | --- |
| Variant | Substrate / soil | Treatment /  specifications | Number  of plants | | Number of pooled  samples analyzed (n) | | Number  of plants = number of samples analyzed (n) | Number  of plants = number of samples analyzed (n) |
| day 0 | peat  substrate | none | 15 | | 5 | | 5 | 0 |
|  |  |  | day7 | day14 | day7 | day14 | Day14 | day56 |
| ARD | ARD soil Heidgraben | fertilized | 15 | 15 | 5 | 5 | 5 | 5 |
| Grass | control soil Heidgraben | fertilized | 15 | 15 | 5 | 5 | 5 | 5 |
| Peat (substrate) | peat substrate + quartz sand (2 + 1) | fertilized | 15 | 15 | 5 | 5 | 5 | 5 |
| Heat | peat substrate + quartz sand (2 + 1) | fertilized, plants 3 days  at 37°C | 15 | 15 | 5 | 5 | 5 | 5 |
| Salt | peat substrate + quartz sand (2 + 1) | one-time application of 50 mL 0.17 M NaCl (10 g L^-1^) at the start of the experiment, fertilized | 15 | 15 | 5 | 5 | 5 | 5 |
| Nutrient starvation | quartz  sand | not fertilized | 15 | 15 | 5 | 5 | 5 | 4 |
| 30 |  | **total** | **195** | | **65** | | **35** | **29** |

PCR efficiency and melting curve analysis should be provided.

Thank you for pointing this out. We will provide this information via the BonaRes database (https://tools.bonares.de/submission/) upon acceptance of the manuscript. <https://www.dropbox.com/sh/rmswsynx6r7elfe/AADCrOYfGY1DnlA1_suLn9Lta?dl=0>

Under conclusions, it is stated that “Their expression was strongly and consistently induced in roots of apple plants grown in three different soils affected by ARD.”. This is in comparison to what? Authors should clearly mention what they are comparing each time. Otherwise, it becomes difficult to understand.

Thank you for pointing this out. We edited the sentence as follows to make the comparison clear: “Their expression was strongly and consistently induced in roots of apple plants grown in three different soils affected by ARD compared to γ-irradiated ARD soil.” (ln. 710 ff.)

Overall, the text should be revised to make it readable and simple.

We are always striving to achieve clarity in the presentation of our research and are thankful for comments on its improvement. We believe that the comments raised by the editor and all reviewers helped greatly to improve the manuscript. If you have further concerns, please let us know.

Thanks a lot for your useful comments and your time!

Editor comments

Dear valued editor,

Thank you for taking the time to help us with our manuscript and your appreciated advice. We removed the “data not shown” in ln 350 of the old manuscript and provided the qPCR data as a supplementary file / DOI, which will be activated upon acceptance via the BonaRes database (https://tools.bonares.de/submission/). Until then, you can access the data under the following link:

<https://www.dropbox.com/sh/rmswsynx6r7elfe/AADCrOYfGY1DnlA1_suLn9Lta?dl=0>

We checked all figures using the PACE tool and improved the resolution for Fig. 2. The reference for Table 3 was added in ln 406 of the revised manuscript. We adjusted the file naming as well as the manuscript formatting according to the provided information.

Thank you for helping us!
