# Supplementary material for: Identification and validation of early genetic biomarkers for apple replant disease
Source: PLoS One. 2020 Sep 24;15(9):e0238876. doi: 10.1371/journal.pone.0238876 (PMC7514092; doi:10.1371/journal.pone.0238876)
Supplement: Attachment — Submitted filename: Response to Reviewers.docx [file pone.0238876.s011.docx]

PONE-D-20-08054
Identification and validation of early genetic biomarkers for apple replant disease
PLOS ONE

Editor: Suprasanna Penna (Academic Editor)

Dear Editor, dear Reviewers,

thank you for your time and support to help us improve this manuscript. We carefully considered the additional questions raised by Reviewer 3. Please again find our responses below indicated in blue below each point. We hope that you will find all points addressed in the right way and look forward to hearing from you again soon.

Thank you and with kind regards

Annmarie-Deetja Rohr and Traud Winkelmann

**REVISION NOTES / DEADLINE 10.09.2020**

**Reviewer #1:**

The comments and questions raised by review have been addressed. Therefore, the current form of manuscript is ready to be accepted for publication.

Thank you! We appreciate your constructive contribution!

**Reviewer #3:**

Instead of stating lie “Apple replant disease (ARD) is a serious threat to producers of apple trees and fruits worldwide.”, you can say Apple replant disease (ARD) is a serious disease in apple which causes ---. Indicate the extent of loss, damage.

Thank for this comment. It is estimated that the fruit yield losses caused by ARD range from 20 – 80 % (Mazzola et al. 2015). Since ARD is a disease complex (Winkelmann et al. 2019) with differing causal agents from site to site the exact losses are difficult to determine. In order to follow your suggestion, we added to line 41 “The estimated yield losses caused by ARD range from 20 – 50 % [6]”.

Basically, the authors need to highlight how BIS3 and B4Hb express under disease condition in comparison to disease-free condition.

Yes, this is exactly what our experiment 1 was aiming at. Since ARD is a disease complex with several pathogens involved we decided against inoculation experiments but chose to work with the affected soil instead. This way, we can represent conditions as close to the field as possible. We used gamma-irradiation to achieve the disease-free condition of the soil and present the comparison that you are asking for in the results of experiment 1. To make this more clear, we added this fact within the objectives in line 115 f.

Also, they need to establish that the expression of BIS3 and B4Hb genes is very specific (express only in the presence of the pathogen).

The results we obtained in this study show that the expression of *BIS3* and *B4Hb* is specific to pathogens present in all three ARD soils and to lesser extent in grass soil. They were not induced in disease-free (disinfected by gamma irradiation) soil, peat substrate and under heat and salt stress or nutrient starvation.

Additional tests with isolated pathogens of the ARD disease complex can help to further investigate the plant’s response, which is beyond this study’s scope. To implement your point, we refer to the new line 761 f. of the conclusions which reads as follows: “Future studies should include expression analyses of the ARD marker genes after inoculation with causal agents that are part of the ARD complex.”

We are aware of studies that investigate the plant response to isolated pathogens involved in the ARD complex such as *Pythium ultimum* (e.g. Zhu et al. 2015, Shin et al. 2016). Zhu et al. observed an upregulation of *BIS* transcription as well in their system as we discuss in lines 670 ff.

Instead of defining the disease in terms of the soils, the authors can define the disease in terms of the presence or absence of the pathogen.
Thank you! ARD has been described for decades and its name and definition have evolved over time. It is true that the term ‘disease’ is misleading here. The most recent definition of ARD is that “ARD describes a harmfully disturbed physiological and morphological reaction of apple plants to soils that faced alterations in their (micro-) biome due to previous apple cultures” (Winkelmann et al. 2019).
Hence, in contrast to classical plant diseases, ARD symptoms are not traced back to the presence of one pathogen. Instead, they are a result of a complex shift in soil microbiota involving both the increase of several pathogens but also changes in beneficial organism communities. Studies have found that this shift depends of the site as well, making the matter very complex.

In order to follow your point, we have added this aspect to the conclusion section stating in line 763 f.: “Furthermore, it would be interesting to correlate gene expression levels to the microbial community composition in the respective ARD soils.”

Is this method of biomarker detection a destructive method?

You are right, with the very young plants we are using in the presented biotests, this method of root sampling is indeed destructive. The aim of this biotest is to sample soil from a field and test it with young plants cultivated for this purpose.

In a transcriptomic study conducted on leaf samples of M26 plants from an identical biotest (Weiß and Winkelmann 2017) we found that *BIS3* and *B4Hb* do not show the same response in the leaves. Further leaf-based candidates for marker genes in the leaves were also not identified in that study.

We are planning to conduct experiments with samples taken from older trees in the field.

We appreciate the thoughts you shared with us and hope that our answers and the corresponding changes in our revised manuscript will improve the understanding.

**Literature cited**

Mazzola M, Strauss SL. Replant disease control and system resilience to pathogen re-infestation in response to Brassica seedmeal amendment. Acta Hortic. 2014: 105–112. doi: 10.17660/ActaHortic.2014.1044.11.

Shin S, Zheng P, Fazio G, Mazzola M, Main D, Zhu Y. Transcriptome changes specifically associated with apple (Malus domestica) root defense response during Pythium ultimum infection. Physiological and Molecular Plant Pathology. 2016; 94: 16–26. doi: 10.1016/j.pmpp.2016.03.003.

Weiß S, Winkelmann T. Transcriptome profiling in leaves representing aboveground parts of apple replant disease affected Malus domestica ‘M26’ plants. Scientia Horticulturae. 2017; 222: 111–125. doi: 10.1016/j.scienta.2017.05.012.

Winkelmann T, Smalla K, Amelung W, Baab G, Grunewaldt-Stöcker G, Kanfra X, et al. Apple replant disease: causes and mitigation strategies. Curr Issues Mol Biol. 2019; 30: 89–106. doi: 10.21775/cimb.030.089.

Zhu Y, Shin S, Mazzola M. Genotype responses of two apple rootstocks to infection by Pythium ultimum causing apple replant disease. Canadian Journal of Plant Pathology. 2016; 38: 483–491. doi: 10.1080/07060661.2016.1260640.
